# Prediction of Quality Substance Content of Hakka Stir-Fried Green Tea Based on Multiple Features of Near-Infrared Spectroscopy

**DOI:** 10.3390/foods15030531

**Published:** 2026-02-03

**Authors:** Yanjiang Qiu, Ting Tang, Jiacheng Guo, Yunfang Zeng, Zihao Li, Qiaoyi Zhou, Dongxia Liang, Caijin Ling

**Affiliations:** 1 Guangdong Provincial Key Laboratory of Tea Plant Resources Innovation and Utilization, Tea Research Institute, Guangdong Academy of Agricultural Sciences, Guangzhou 510640, China; 2 Faculty of Innovation Engineering, Macau University of Science and Technology, Macau 999078, China; 3 College of Engineering, South China Agricultural University, Guangzhou 510642, China

**Keywords:** near-infrared spectroscopy, Hakka stir-fried green tea, adaptive Fourier decomposition, multiple feature variables, regression model

## Abstract

The contents of biochemical components, such as theanine, tea polyphenols, water extract, and soluble sugar in Hakka stir-fried green tea (HSGT), serve as important indicators reflecting the intrinsic quality of tea leaves. In this study, 171 HSGT samples are collected, and their near-infrared spectroscopy (NIRS), together with the contents of the four indicators, are determined. The aim is to establish prediction models for these four indicators by extracting multiple features from the NIRS data. First, the NIRS data is preprocessed. Then, multiple features are extracted using competitive adaptive reweighted sampling (CARS), adaptive Fourier decomposition (AFD), fast Fourier transform (FFT), continuous wavelet transform (CWT), and band combination (BC). Finally, ridge regression (RR) and partial least squares regression (PLSR) models are constructed based on the NIRS features to predict the four indicators. Experimental results show that the model combining multiple features, namely CARS + AFD + BC, delivers the best overall performance. Specifically, the RR model based on multiple features provides the most accurate predictions for theanine, tea polyphenols, and soluble sugar, while the PLSR model performs better for water extract. This study provides a rapid and accurate method for detecting the substance content in HSGT.

## 1. Introduction

Hakka stir-fried green tea (HSGT), also known as Hakka stir-fried tea, or Hakka green tea, is one of the most representative traditional teas in Guangdong province, China [1]. This tea is primarily distributed in the core Hakka settlements (a Han Chinese subgroup), including Meizhou, Heyuan, and Huizhou in Guangdong province, and is therefore referred to as Hakka stir-fried green tea [1,2]. The unique quality characteristics of traditional stir-fried green tea are formed during its processing. Its sensory quality is a comprehensive reflection of the composition and proportion of its internal components. Among the quality-related substances in traditional stir-fried green tea, tea polyphenols offer numerous benefits, including antioxidant and blood pressure-lowering effects [3]. Theanine, a bioactive compound, exhibits anti-inflammatory, neuroprotective, and metabolic regulatory properties [4]. Soluble sugar is an important sweetening component in tea soup, which can mitigate the bitter taste caused by tea polyphenols and caffeine [5]. Water extract, a methylxanthine alkaloid, exhibits effects such as promoting alertness, refreshing the mind, diuresis, and strengthening the heart [6]. Therefore, the content of major biochemical components, such as theanine, tea polyphenols, soluble sugar, and water extract, is an important indicator of the inherent quality of HSGT leaves [1,7]. However, research on the substance of HSGT remains limited. Additionally, traditional chemical methods such as gas chromatography are time-consuming, labor-intensive, and require skilled technicians to operate when detecting the content of these important indicators [8].

Due to its high penetration ability, near-infrared spectroscopy (NIRS) can penetrate the surface of samples and analyze opaque materials. With its broad absorption peaks, NIRS can be used to simultaneously detect multiple chemical components, making it widely used for the quantitative and qualitative analysis of finished tea quality [9,10]. Ren et al. proposed a multivariate selection strategy based on variable space optimization from large to small, and quickly evaluated the tenderness and ranking of black tea using NIRS technology [11]. The results showed that the IGA-PSO-SVM model based on radial basis functions selected four feature variables in the prediction process, and its correct discrimination rate (CDR) was 95.28%, indicating excellent prediction performance. However, the variables selected by this strategy are only the reflectance corresponding to the characteristic bands, and the effective information contained is not sufficiently comprehensive.

Vegetation indices can be used to obtain quantitative and qualitative measurements of biophysical and biochemical parameters of vegetation, including embedded content [12]. Most widely used vegetation indices today are combinations of visible and NIR wavelengths. Additionally, vegetation index parameters can be tailored to specific analytical objectives to enhance the coefficient of determination ($R^{2}$) of the estimation model [13,14]. Duan et al. constructed an estimation model for theanine based on an optimized vegetation index, achieving $R^{2}=0.81$, which enables accurate quantification of theanine [15]. Additionally, the transformation coefficient of spectral signals provides multi-scale time-frequency representations, demonstrating superior denoising capabilities and feature compression efficiency, thereby enabling the extraction of localized spectral signatures and high-resolution diagnostic features [16]. The high-frequency feature coefficients obtained through the wavelet transform are widely used to extract effective detail information [17]. Jiang et al. mined high-frequency feature bands using continuous wavelet transform (CWT) and integrated them with sensitive hyperspectral vegetation index features [18]. Based on this, a random forest regression model was developed to estimate annual biomass increment and foliage nitrogen content in tea plantations. The model integrating multi-source features achieved $R^{2}=0.68$ for nitrogen content estimation, outperforming the single-feature model ($R^{2}=0.52$). This indicates that the combined features contain more effective information and can improve the stability of the model. The spectral information corresponding to the feature bands selected by algorithms can effectively reflect the biophysical and biochemical features of the target [19]. Sun et al. employed the competitive adaptive re-weighted sampling (CARS) algorithm and stepwise projection method to screen feature band information, and constructed a quantitative analysis model for tea moisture content using the selected spectral data [20]. The results demonstrate that the combined algorithm of SG-MSC and CARS-SR achieved the best predictive performance, yielding a mixed logistic regression model with $R^{2}=0.863$.

In this study, various preprocessing methods are employed to process the NIRS data. Subsequently, multiple NIRS features are extracted from three different perspectives, including band combination (BC), data decomposition, and band selection. Based on the obtained multiple features, prediction models are established for the substance content of theanine, tea polyphenols, water extract, and soluble sugar in HSGT. The contributions of this article can be summarized as follows:

(1) This study measures the NIRS data of 171 HSGT samples, along with their contents of theanine, tea polyphenols, water extract, and soluble sugar, with the aim of establishing the relationship between NIRS and the contents of the four different substances.
(2) A combination of preprocessing techniques and mathematical transformations is utilized to process NIRS data. First, four methods, including Savitzky Golay (SG) smoothing, multivariate scatter correction (MSC), standard normal variate (SNV), detrended term (DT), and moving average (MA), are applied to preprocess the near-infrared spectral data. Then, the processed data are mathematically transformed using the first derivative (FD) and second derivative (SD).
(3) This study proposes extracting NIRS features from multiple perspectives to more comprehensively extract useful information from NIRS. First, based on the correlation between NIRS and different substance indicators, six distinct spectral index calculation methods are derived. Second, from the perspective of data decomposition, adaptive Fourier decomposition (AFD), CWT, and fast Fourier transform (FFT) are utilized to decompose the NIRS data and extract the corresponding feature coefficients. Moreover, CARS is employed to screen feature bands from the NIRS data.
(4) Ridge regression (RR) and partial least squares regression (PLSR) models are established for the four substance indicators using different NIRS features, respectively. The results reveal that the model constructed with multiple features, namely CARS + AFD + BC, demonstrated the best performance for all four indicators. Furthermore, the RR model is identified as the optimal model for theanine, tea polyphenols, and soluble sugar, while the PLSR model is the best for water extract.
(5) It is worth noting that this study is the first to apply AFD to NIRS data decomposition. AFD can sparsely represent the original NIRS data in a functional form, enabling the extraction of NIRS features without information loss. The experiments demonstrate that the feature coefficients extracted using AFD are more effective for detecting the substance content of HSGT compared to traditional methods, such as CWT and FFT. Furthermore, AFD holds significant potential for data mining applications in other crops.

## 2. Materials and Methods

**Figure 1:**
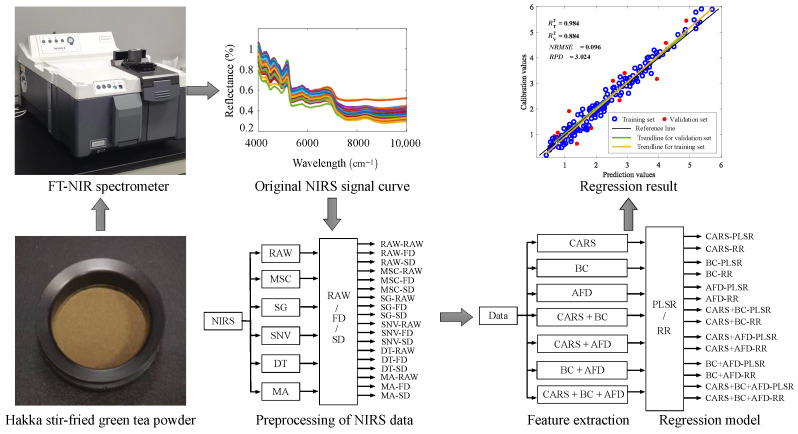
Process of establishing the model for predicting the quality substance content of HSGT based on the NIRS features.

The framework proposed in this study for predicting the quality substance content of HSGT is illustrated in Figure 1. The framework comprises four key stages: sample and data acquisition, data preprocessing, feature extraction, and model construction and evaluation. Each of these stages is elaborated in detail in this section.

### 2.1. Sample and Data Acquisition

#### 2.1.1. Sample Acquisition

HSGT belongs to the category of long-fried green tea. It is a green tea product made from the buds and leaves of small- and medium-leafed tea trees cultivated in the Hakka region of Guangdong province. The tea is processed through withering, greening, kneading, and stir-frying [21]. The HSGT samples used in this study were sourced from the core production areas of Xianhu Mountain and Wuhua and were packaged in sealed bags after processing. The samples comprise two types: semi-fried and fully fried, totaling 171 samples.

#### 2.1.2. Data Acquisition

The HSGT samples were ground, sieved, and then analyzed using an FT-NIR spectrometer (Thermo Electron Co., Waltham, MA, USA) in diffuse reflectance mode to obtain their NIRS data. Each sample (15 g) was collected three times, and finally, the spectral information of the corresponding sample was represented by the average spectral data obtained from the three acquisitions. The tea polyphenol content was determined using the Folin phenol method according to GB/T 8313-2008 [22]. The total amount of free amino acids was determined using the ninhydrin colorimetric method, in accordance with GB/T 8314-2013 [23]. The water extract content was measured by the total analytical procedure according to GB/T 8305-2013 [24]. The soluble sugar content was determined using the anthrone colorimetric method, according to GB/T 5009.8-2016 [25]. The process of extracting the content of four different substances from HSGT in this study can be summarized as follows:

(1) Tea polyphenols. First, the tea samples are ground and extracted with 70% (*v*/*v*) methanol in a water bath at 70 degrees Celsius to obtain the tea polyphenol extract. Subsequently, the extract (1.0 mL) is mixed with the Folin-Ciocalteu reagent (5.0 mL). Then, a sodium carbonate solution is added to create an alkaline environment. Under this condition, the phenolic hydroxyl groups reduce the heteropolyphosphotungstate-molybdate complexes, resulting in the formation of a stable blue chromophore. Finally, the absorbance of the solution is measured at a wavelength of 765 nm. The total tea polyphenol content is calculated by comparing the absorbance to a standard curve prepared with gallic acid of known concentrations.
(2) Free amino acids. The tea infusion (1.0 mL) is mixed with ninhydrin reagent (0.5 mL). Then, the mixture is heated in a boiling water bath for a defined period (15 min) to facilitate the color development reaction. During this process, the free amino acids react with ninhydrin under slightly acidic conditions to form a purple chromophore. After cooling to room temperature, the solution is diluted to a predetermined volume (25 mL) with water. The absorbance of the resulting solution is then measured at a wavelength of 570 nm. The concentration of free amino acids in the sample is quantified by comparing the absorbance against a standard curve prepared with a known amino acid.
(3) Water extract. The tea samples are first ground and passed through a specified sieve. A portion of the sample is accurately weighed into a pre-weighed crucible and dried to constant weight at 105 ± 2 degrees Celsius to determine the dry matter content. Subsequently, the dried sample is transferred to a conical flask and extracted with boiling distilled water for a defined period under reflux condensation to prevent solvent loss. The extract is then filtered through a pre-dried and weighed filter paper. The residue along with the filter paper is thoroughly washed, dried again to constant weight at 105 ± 2 degrees Celsius, and weighed. The water extract content is calculated as the mass loss of the sample after extraction, expressed as a percentage of the original dry mass of the sample.
(4) Soluble sugars. Soluble sugars are extracted from the sample with hot water. An aliquot of the resulting extract is mixed with anthrone reagent, followed by the rapid addition of concentrated sulfuric acid. The mixture is heated in a boiling water bath for a precise duration. During heating, the carbohydrates are dehydrated by the acid to form furfural derivatives, which condense with anthrone to yield a blue-green chromophore. The absorbance of the cooled solution is measured at a wavelength of 620 nm using a spectrophotometer. The soluble sugar concentration is determined by interpolating the absorbance value against a standard curve prepared with glucose treated identically.

### 2.2. Data Preprocessing Methods

Since the samples originate from different tea processing factories, variations in processing methods and other factors may lead to abnormal levels of certain indicators. Therefore, the box plot method is first applied to eliminate sample data with anomalous values for each indicator. Additionally, the original NIRS signal typically contains noise and redundant information. To enhance the stability and accuracy of the prediction model for indicator content, it is essential to preprocess and analyze the NIRS signal [26].

**Figure 2:**
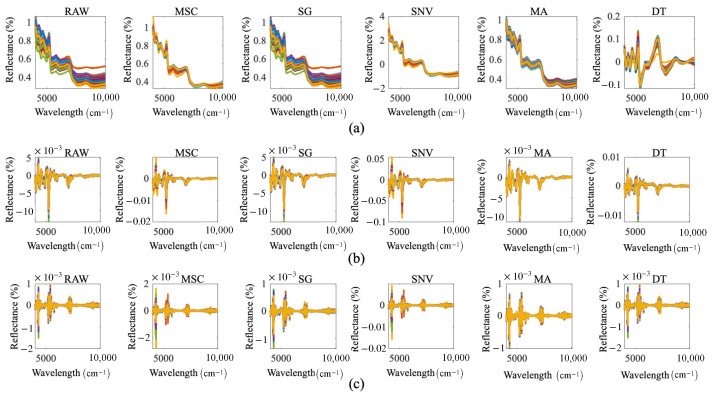
NIRS curves after preprocessing: (**a**) Curves without derivative transformation; (**b**) FD-processed curves; (**c**) SD-processed curves.

Common NIRS preprocessing methods include SNV, MA, SG, MSC, and DT [27,28]. SNV is capable of mitigating the effects of solid particle size, surface scattering, and optical range variations on NIRS, effectively eliminating baseline drift and intensity variations in spectral data [29,30]. MA reduces random noise in NIRS by averaging the data within a specified window, which improves the signal-to-noise ratio of the sample signal [31]. SG works by fitting local spectral trends and removing noise components that deviate from the trend. It focuses on removing localized noise while preserving subtle reflectance differences in the NIRS data [32]. MSC takes the mean value of all spectral data as the reference spectrum, performs linear regression on each measured spectrum, and then calculates the corrected spectrum. This method can effectively distinguish the scattered chemical signals in the spectral data and reduce the interference of total reflection and diffuse reflection on the spectral model [33]. DT is designed to remove baseline offsets and curvature from spectral signals [34]. In addition, derivative transformation can effectively reduce noise interference. It not only mitigates the baseline effect but also enhances the spectral features [35]. Therefore, in this study, the raw NIRS data of HSGT samples are preprocessed using SNV, MA, SG, MSC, and DT, respectively. Subsequently, the preprocessed data are subjected to FD and SD, respectively. The transformed NIRS curves are illustrated in Figure 2, where RAW denotes the raw data.

### 2.3. Feature Extraction Methods

#### 2.3.1. Band Combination

When combining arbitrary NIRS bands within the given band range, it is essential to consider both the multidimensional relationships among spectral features and the mutual interactions between NIRS bands. This approach significantly enhances the extraction of hidden spectral information [35]. Such BC methodology enables more comprehensive characterization of spectral features, and through it, we can extract NIRS features exhibiting stronger correlations with the target indicator content. Based on the NIRS data, this article proposes using six spectral indices, namely sample ratio (SR), normalized SR (NSR), difference spectral index (DSI), normalized DSI (NDSI), generalized DSI (GDSI), and transformed NDSI (TNDSI), to calculate and obtain the effective BC features for each indicator. The final features are selected based on the absolute values of their Pearson correlation coefficients (PCCs) with each indicator, as these values are relatively high. The calculation of these six spectral indices varies depending on the specific indicator. The corresponding calculation formulas are listed in Table 1, where $R_{m}$ denotes the reflectance corresponding to band *m*.

#### 2.3.2. Feature Band Screening

Due to the high autocorrelation of reflectance in the original NIRS bands, where some bands exhibit similar or redundant reflectance patterns, it is necessary to screen the NIRS data and select the most representative and informative bands for subsequent analysis [36,37]. After comparing three feature band selection methods, such as the successive projections algorithm (SPA), uninformative variable elimination (UVE), and CARS, this study ultimately selected CARS for feature band screening.

#### 2.3.3. Feature Coefficient Extraction

AFD is a method proposed by Qian’s team to analyze non-stationary signals [38]. The purpose of using AFD to decompose the reflectance is to transform the NIRS data into the AFD domain for analysis, which helps to uncover hidden information in the data without losing information. Next, we will provide a detailed introduction to AFD-based features.

For an arbitrary NIRS signal, it is energy-limited and can be expressed as

(1) $$x\left(t\right)=\sum_{k=-\infty }^{+\infty }c_{k}e^{ikt},\sum_{k=-\infty }^{+\infty }{\left|c_{k}\right|}^{2}<+\infty ,$$

where *t* represents the wavelength transformed to the range of [0,2π], and the Fourier coefficient $c_{k}=\langle x,e^{ikt}\rangle =\frac{1}{2\pi }\int_{0}^{2\pi }x\left(t\right)e^{-ikt}dt$. Let C be the complex plane, and $D=\left\{z=re^{it}\in C:0⩽r<1\right\}$ denotes an open unit disc centered at the origin in C. The complex Hardy space on D can be labeled as $H^{2}\left(D\right)$. Let T denote the boundary of D, and $x\left(t\right)$ can be transformed into the Hilbert space $L^{2}\left(T\right)$, that is,

(2) $$x\left(t\right)=\sum_{k=-\infty }^{+\infty }c_{k}e^{ikt}≜f\left(e^{it}\right).$$

Due to the symmetry of the Fourier spectrum, $f\left(e^{it}\right)$ can be rewritten as

(3) feit=∑k=−∞0ckeikt+∑k=0+∞ckeikt−c0≜f−eit+f+eit−c0=2Ref+eit−c0,

where $f^{-}$ and $f^{+}$ denote the negative and positive frequency parts of *f*, and Re· represents taking the real part of function. $f^{+}$ can be obtained through

(4) $$f^{+}\left(e^{it}\right)=\frac{1}{2}\left(f\left(e^{it}\right)+iHf\left(e^{it}\right)+c_{0}\right),$$

where $Hf\left(e^{it}\right)$ means performing the Hilbert transform on $f\left(e^{it}\right)$.

AFD is based on the Takenaka-Malmquist (TM) system in $H^{2}\left(D\right)$, which is denoted as ${\left\{B_{k}\left(e^{it}\right)\right\}}_{k=1}^{\infty }$ with

(5) $$B_{k}\left(e^{it}\right)=\frac{\sqrt{1-|a_{k}{|}^{2}}}{1-{\bar{a}}_{k}e^{it}}\prod_{j=1}^{k-1}\frac{e^{it}-a_{j}}{1-{\bar{a}}_{j}e^{it}},a_{j}\in D,$$

where ${\bar{a}}_{j}$ represents the complex conjugate of $a_{j}$. $B_{k}\left(e^{it}\right)$ is called the *k*-order weighted Blaschke product. The evaluator $e_{a_{k}}\left(e^{it}\right)=\frac{\sqrt{1-|a_{k}{|}^{2}}}{1-{\bar{a}}_{k}e^{it}}$, which is the $L^{2}$-norm normalized Szegö kernel at $a_{k}$. For arbitrary $f^{+}\left(e^{it}\right)\in H^{2}\left(D\right)$, after being decomposed into *K* steps by AFD, we can obtain

(6) $$f^{+}\left(e^{it}\right)=\sum_{k=1}^{K}d_{k}B_{k}\left(e^{it}\right)+\gamma_{K},$$

where the *k*-th AFD coefficient $d_{k}=\langle f_{k}^{+},e_{a_{k}}\rangle =\frac{1}{2\pi }\int_{0}^{2\pi }f^{+}\left(e^{it}\right){\bar{e}}_{a_{k}}\left(e^{it}\right)dt$, and the standard error $\gamma_{k}$ is given by

(7) $$\begin{array}{cc} \gamma_{k} & =f_{k+1}^{+}\left(e^{it}\right)\frac{e^{it}-a_{k}}{1-{\bar{a}}_{k}e^{it}},\\ f_{k+1}^{+}\left(e^{it}\right) & =\frac{f_{k}^{+}\left(e^{it}\right)-\langle f_{k}^{+},e_{a_{k}}\rangle e_{a_{k}}\left(e^{it}\right)}{\frac{e^{it}-a_{k}}{1-{\bar{a}}_{k}e^{it}}}. \end{array}$$

The parameter $a_{k}$ for the *k*-th decomposition is selected using the maximal selection principle, namely,

(8) $$a_{k}=\arg\max\left\{{\left|\langle f_{k}^{+},e_{a}\rangle \right|}^{2}:a\in D\right\}.$$

**Figure 3:**
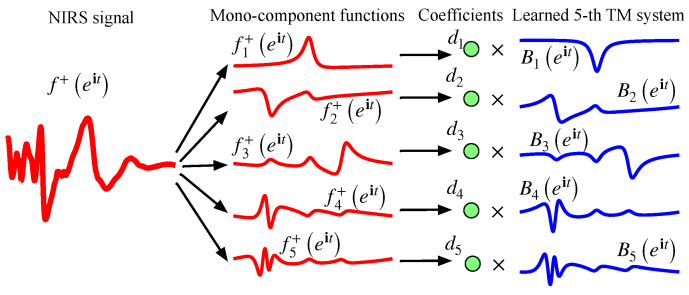
Framework of the AFD-based feature extraction.

After $f^{+}\left(e^{it}\right)$ is decomposed into *K* terms by AFD, we obtain the learned *K*-TM system ${\left\{B_{k}\left(e^{it}\right)\right\}}_{k=1}^{K}$ and *K* coefficients ${\left\{d_{k}\right\}}_{k=1}^{K}$. Modulus of coefficients ${\left\{|\langle f_{k}^{+},e_{a_{k}}\rangle {|}^{2}\right\}}_{k=1}^{K}$ are defined as the AFD-based features of the NIRS signal. Figure 3 visually illustrates the decomposing process of AFD with 5 levels. As shown in Figure 3, the AFD coefficient is the value obtained by projecting the NIRS signal onto the TM system, which reflects the energy information of different components in the signal.

In the experiment, we used the relative energy error (REE) as the fitting degree of the AFD algorithm, which is expressed as

(9) $$\mathrm{REE}=\frac{∥\tilde{f}-f∥}{∥f∥},$$

where $\tilde{f}$ is the approximation of *f*. To ensure that valid information is not lost, we set the REE of all samples to 0.995 for AFD analysis. Assume that the maximum decomposition level among all samples is $N_{\mathrm{AFD}}$. Therefore, AFD decomposition with a fixed decomposition level $N_{\mathrm{AFD}}$ is performed on all samples to further obtain AFD-based features.

**Figure 4:**
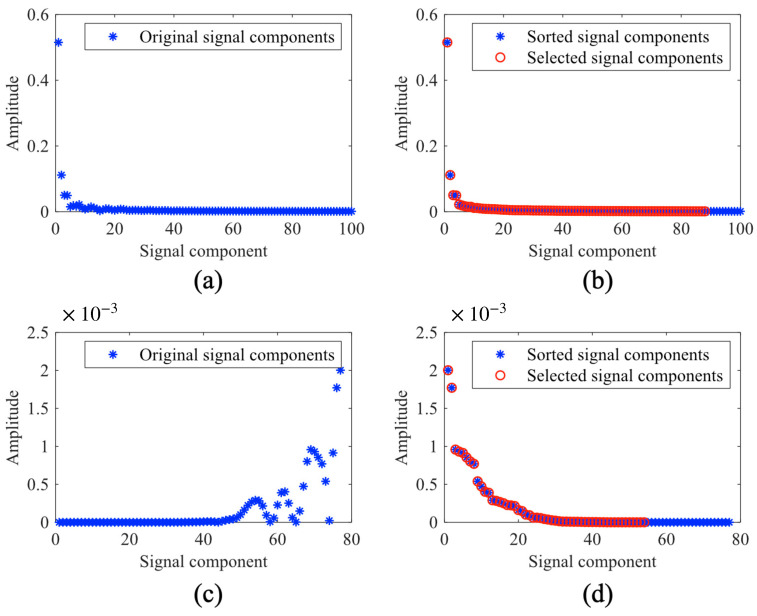
Feature extraction using FFT and CWT: (**a**) Original signal obtained by FFT; (**b**) Feature coefficients obtained by FFT; (**c**) Original signal obtained by CWT; (**d**) Feature coefficients obtained by CWT.

After decomposing the NIRS signals into different components using AFD, the energy values of these components are used to characterize the original signal. This approach is conceptually similar to FFT-based NIRS analysis but differs in the basis functions employed for signal projection [39]. For comparative analysis, FFT is employed to extract energy values from different frequency components in NIRS signals. First, FFT is computed for all samples (Figure 4a). The resulting energy values are then sorted in descending order, and amplitudes accounting for 90% cumulative energy are selected as FFT-based features (Figure 4b). To maintain feature number consistency across samples, the minimal feature count ($N_{\mathrm{FFT}}$) among all samples is adopted as the uniform feature dimension. Consequently, the highest $N_{\mathrm{FFT}}$ energy values from each sample are selected as the FFT-based features for the NIRS signal.

In addition, CWT is also a commonly used signal analysis method. With its inherent time-frequency localization capability, CWT facilitates multi-scale analysis while effectively separating signal from noise. The availability of diverse wavelet basis functions enables CWT to reveal subtle spectral structures and capture complex spectral variations. These features assist researchers in better understanding and interpreting intricate spectral information [40,41]. Therefore, we also incorporate CWT-based features for comparative analysis. The preprocessed NIRS signals are transformed using CWT with three wavelet bases (Morse, Amor, and Bump) to obtain their scale coefficients. To maintain feature dimensionality consistency across samples, the top $N_{\mathrm{CWT}}$ coefficients from each sample are selected as CWT-based features (Figure 4c,d), where $N_{\mathrm{CWT}}$ represents the minimal coefficient count observed among all samples.

### 2.4. Model Construction and Evaluation

Following feature extraction, PLSR and RR are employed to develop prediction models for the content of theanine, tea polyphenols, water extract, and soluble sugar in HSGT. PLSR integrates principles from multiple linear regression, principal component analysis (PCA), and canonical correlation analysis. This combined approach enables dimensionality reduction while maximizing information extraction from the data, effectively addressing both collinearity and nonlinearity among features, while elucidating their interrelationships [42,43]. These characteristics make PLSR particularly suitable for analyzing NIRS data. The RR model has been widely used in spectral data estimation due to its ability to handle multicollinearity while maintaining strong interpretability and computational efficiency [44]. In this study, the experiment employed ten-fold cross-validation, with each experiment repeated across 50 Monte Carlo simulations. Final results represent the average of all simulation outcomes.

To comprehensively evaluate the model performance, we employed three metrics: the coefficient of determination ($R^{2}$) for both training and validation sets, the normalized root mean square error (NRMSE) of the validation set, and the ratio of performance to deviation (RPD) of the validation set. These metrics are calculated as follows:

(10) $$R^{2}=1-\frac{\sum_{i=1}^{n}{\left(y_{i,a}-y_{i,p}\right)}^{2}}{\sum_{i=1}^{n}{\left(y_{i,a}-{\bar{y}}_{a}\right)}^{2}},$$

(11) $$NRMSE=\frac{\sqrt{\sum_{i=1}^{n}{\left(y_{i,a}-y_{i,p}\right)}^{2}/n}}{\sum_{i=1}^{n}y_{i,a/n}},$$

(12) $$RPD=\frac{\sqrt{\sum_{i=1}^{n}{\left(y_{i,a}-{\bar{y}}_{p}\right)}^{2}/\left(n-1\right)}}{\sqrt{\sum_{i=1}^{n}{\left(y_{i,a}-y_{i,p}\right)}^{2}/n}},$$

where *n* is the number of samples; $y_{i,a}$ and $y_{i,p}$ represent the measured and predicted values of the indicator for the *i*-th sample, respectively. ${\bar{y}}_{a}$ and ${\bar{y}}_{p}$ denote the mean of the measured values and the predicted values of the indicator, respectively. The model performance can be interpreted as follows: (1) $R^{2}$ values closer to 1 indicate a better model fit. (2) Lower NRMSE values correspond to higher inversion accuracy. (3) Larger RPD values reflect stronger predictive capability.

## 3. Results and Discussion

### 3.1. Preprocessing and Feature Extraction Results

Spectral indices constructed by combining different NIRS bands can enhance certain hidden information in NIRS [45]. In order to obtain the results of BC that are closely related to the indicators, after processing the NIRS data using 18 preprocessing methods, the BC features of theanine, tea polyphenols, water extract, and soluble sugar are screened out using PCC. The study used a dataset comprising 171 samples. The significant PCC is 0.196 when p<0.01. From Table 1, the correlation coefficients of the BC features for four indicators are greater than 0.196, which is a highly significant correlation.

**Figure 5:**
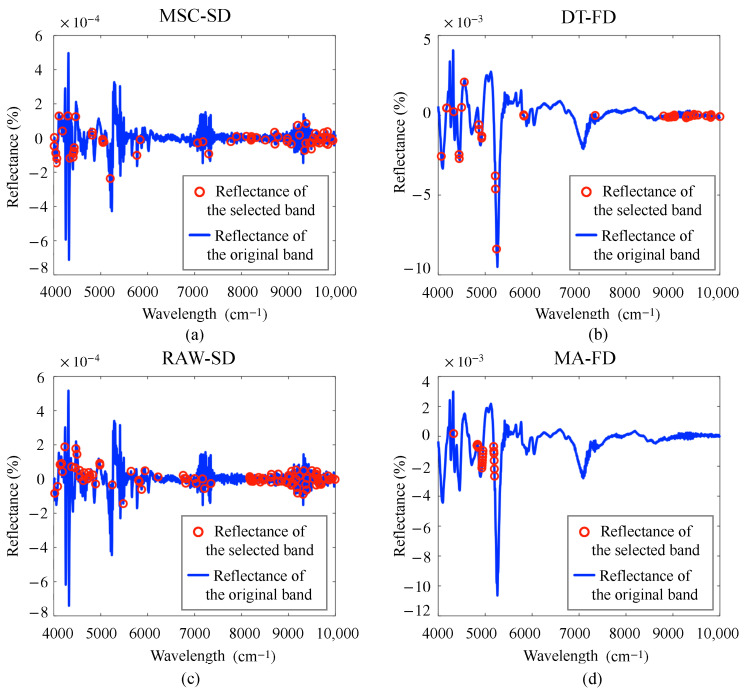
Results of band screening of NIRS by CARS under different indicates: (**a**) Theanine; (**b**) Tea polyphenols; (**c**) Soluble sugar; (**d**) Water extract.

Figure 5 presents the optimal band selection results obtained from CARS-extracted features for each target indicator, corresponding to the best performing models. The detailed band ranges are listed in Table 2, along with comparative results from other studies for these four indicators. By analyzing the results in Table 2, we observe that most feature bands screened in this study overlap with those identified in existing studies, confirming the reliability of our screening method. Deviations may arise from moisture in tea powder affecting absorption regions of O-H bond bending vibrations and C-H bond stretching deformation reflections [46], or differences in sample size and substance content of HSGT.

Besides, it is worth noting that the AFD-based features are not tied to specific NIRS bands but instead correlate with the NIRS curves of HSGT powder samples specifically, the energy values derived from projecting NIRS onto the TM system (see Section 2.3.3).

### 3.2. Prediction of Four Indicators Under Different Features

As previously noted in Section 2.3, the features employed are extracted using three distinct techniques: CARS, BC, and AFD. Accordingly, feature sets are categorized as single-feature (containing one technique), dual-feature (combining two different techniques), and triple-feature (combining all three techniques). The predictive performance of the optimal models based on these feature categories for the four target indicators is summarized in Table 3, where $R_{T}$, $R_{\mathrm{Tp}}$, $R_{\mathrm{Ss}}$, and $R_{\mathrm{We}}$ represent the preprocessing methods of theanine, tea polyphenols, soluble sugar, and water extract in Table 1, respectively. We analyze the results in Table 3 with respect to three key aspects: features, preprocessing methods, and models.

Regarding feature selection, the model based on the triple feature CARS + BC + AFD achieved the best predictive performance in most cases. Specifically, for the tea polyphenol content, the model based on the triple feature attains an $R_{\mathrm{validate}}^{2}$ of 0.606, which is significantly higher than those of all models based on the single feature (0.541, 0.351, 0.513) and based on the dual feature (0.526, 0.582, 0.476). For soluble sugars, the model based on the triple feature yields the highest $R_{\mathrm{validate}}^{2}$ of 0.805, among all combinations, along with well-balanced and excellent NRMSE and RPD values. Similarly, for the water extract content, the model based on the triple feature also produces the highest $R_{\mathrm{validate}}^{2}$ of 0.561. In the case of theanine, although the $R_{\mathrm{validate}}^{2}$ of the triple-feature-based model is slightly lower than that of the model based on the dual-feature CARS + AFD, it remains markedly superior to all other single- and dual-feature combinations. Furthermore, its $R_{\mathrm{train}}^{2}$ is the highest, indicating that the model has an excellent fit and stable performance.

Based on the results of Table 3, the model based on the BC-based features consistently demonstrated the weakest predictive capability. This limitation arises because the formulation of spectral indices inherently discards a significant portion of the spectral data. As noted in prior studies, the eliminated components may include not only redundant noise, but also potentially useful information [50,51]. This finding indicates that relying exclusively on simple algebraic combinations of bands provides insufficient information for constructing robust prediction models. In contrast, the models based on AFD exhibit considerable instability, with their validation performance $R_{\mathrm{validate}}^{2}$ fluctuating widely, from 0.364 to 0.520 across indicators. This variability suggests that while AFD-based features contain useful information, their use in isolation yields an incomplete and potentially noisy representation of the target chemical properties. Conversely, the CARS-based model consistently delivers the strongest and most stable performance among the single-feature approaches. This superiority is attributed to its unique variable selection mechanism. The CARS algorithm iteratively constructs PLSR models, eliminates wavelengths with smaller absolute regression coefficients, and ultimately selects the feature subset that minimizes the root-mean-square-error of cross-validation. This process ensures that the retained bands exhibit a strong correlation with the target indicators [52]. An analysis of dual-feature combinations reveals significant interaction effects and a notable short-board effect. The combination of CARS and BC (CARS + BC) shows minimal, or even negative, improvement compared to using CARS alone. This result implies that adding basic spectral indices after key wavelength selection contributes little new information and may introduce redundancy. Similarly, the BC + AFD combination performs poorly across all indicators with $R_{\mathrm{validate}}^{2}$ from 0.444 to 0.577, underscoring that without the foundation of spectrally screened information by CARS, the fusion of other feature types fails to achieve meaningful synergy. The consistently superior dual-feature combination is CARS + AFD. This result highlights a critical finding that the fusion of key spectral information by CARS with coefficient information by AFD generates a significant synergistic effect, leveraging complementary data representations. Ultimately, the triple-feature combination, CARS + BC + AFD, can be viewed as an enhanced and stabilized extension of the powerful pair CARS + AFD. By incorporating BC, which may contribute broader spectral contextual information, this combination achieves a more balanced and comprehensive feature set. Consequently, it delivers the most robust and optimal performance across multiple evaluation metrics.

The preprocessing of NIRS data exerts a significant influence on model performance, with the optimal method being highly dependent on the target indicator. For theanine, the combination of MSC and SD yields the best results. In contrast, models for tea polyphenols and water extract content benefit more from DT-FD, and MA-FD, respectively. For soluble sugars, applying a second derivative to the raw spectrum (RAW-SD) is sufficient to achieve strong performance. This dependence aligns with the fundamental principle of NIRS analysis, namely, distinct chemical components exhibit unique spectral response characteristics. Therefore, effective preprocessing must be tailored to mitigate specific interferences and enhance the relevant spectral signatures for each analyte. The model selection also shows a strong correlation with the feature set employed. RR is the predominant and more effective choice in most scenarios, particularly when utilizing CARS-selected or fused features. PLSR, however, is more suitable to models using only the BC or AFD-based features, as well as some combinations of water extract. This pattern suggests that the inherent dimensionality reduction and supervised feature extraction capabilities of PLSR become advantageous when the initial feature information is weaker or exhibits a distinct collinearity structure. In summary, when the feature set is information-rich and of high quality, such as after CARS screening or strategic fusion, the simpler regularized regression (RR) is often adequate to build an excellent model. Conversely, PLSR appears more suitable when dealing with limited feature information or pronounced multicollinearity. To substantiate the conclusions drawn from Table 3, comprehensive experimental results are provided in Tables S1 and S2 in Supplementary Materials.

### 3.3. Comparison of Prediction Results Under Different Feature Coefficient Extraction Methods

To compare with the AFD-based feature, this study employed CWT-based and FFT-based features to construct estimation models for four indicators of HSGT. Table 4 presents the optimal model results constructed by CWT-based and FFT-based features, where Amor, Morse, and Bump represent three types of wavelet basis functions, respectively.

**Figure 6:**
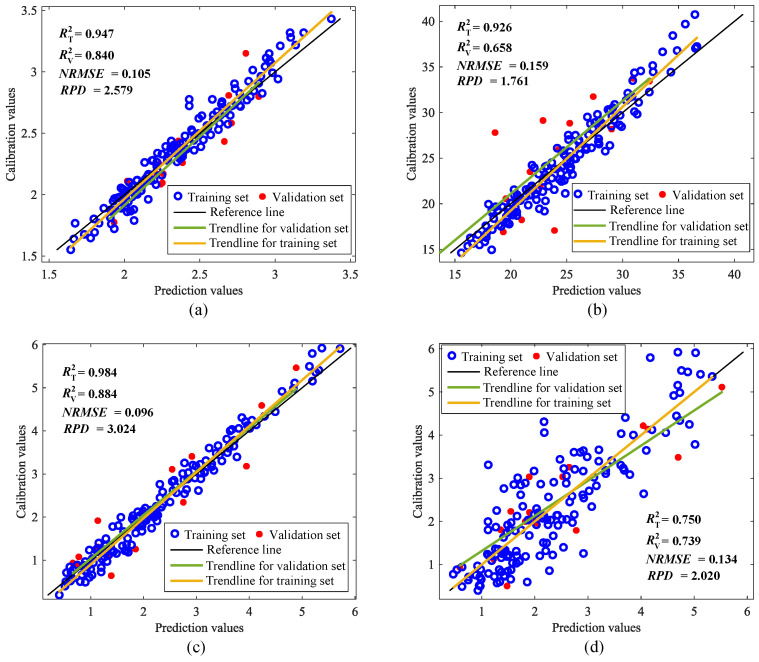
Results of the optimal model constructed by CARS + BC + AFD under different indicators: (**a**) Theanine; (**b**) Tea polyphenols; (**c**) Soluble sugar; (**d**) Water extract.

As can be seen in Table 4, the $R_{\mathrm{validate}}^{2}$ of the models based on FFT or various wavelets (Bump, Amor, Morse) is generally low and highly unstable. For theanine, the values are only 0.399 for FFT and 0.392 for Bump; for water extracts, CWT achieves a slightly higher value of 0.529. Overall, none of the conventional signal decomposition methods demonstrate the comprehensive potential shown by AFD across all indicators. Regarding the dual-feature combinations, Table 3 shows that the CARS + AFD pairing delivers excellent performance, with $R_{\mathrm{validate}}^{2}$ of 0.780 for theanine, 0.582 for tea polyphenols, and 0.802 for soluble sugars. In contrast, Table 4 indicates that replacing AFD with other decomposition methods leads to a consistent decline in performance. Based on this, a conclusion can be drawn that the key spectral features obtained by AFD synergize more effectively with those from CARS. The adaptive nature of AFD better captures local frequency domain patterns associated with specific chemical components, whereas fixed-basis methods such as FFT and CWT may introduce irrelevant noise or fail to optimally match the signal structure. As shown in Table 3, the triple-feature combination CARS + BC + AFD achieves the $R_{\mathrm{validate}}^{2}$ values of 0.770, 0.606, 0.805, and 0.561 across the four indicators, performing optimally on multiple indicators. In Table 4, none of the triple-feature combinations, such as CARS + BC + FFT or CARS + BC + DWT, fully surpass or match this performance. This comparison conclusively demonstrates that augmenting the informative CARS + BC spectral data with AFD-based features yields performance gains that cannot be attained by incorporating FFT or CWT-based features. The optimal prediction results of the estimation model constructed by CARS + BC + AFD for these four indicators are shown in Figure 6. To substantiate the conclusions drawn from Table 4, comprehensive experimental results are provided in Tables S3 and S4 in Supplementary Materials.

The CWT-based features are generated by decomposing NIRS into scale coefficients through CWT. Although all wavelet bases produce the same number of coefficients for a given spectrum, the information content varies substantially across different basis functions. Our results identify which wavelet basis (Amor, Morse, or Bump) generated the most effective features for each indicator. The CWT-based features partially preserve the information from raw NIRS data. However, their fixed wavelet basis functions may fail to capture certain subtle spectral features. In contrast, FFT-based features represent the amplitude values of dominant frequency components after energy-based sorting. While FFT provides frequency-domain representations of NIRS data, it lacks the inherent capability to discriminate between chemically relevant signals and noise. There are fundamental differences between these approaches: FFT decomposes NIRS data into fixed sine functions; CWT utilizes predefined wavelet basis functions; AFD adaptively generates optimal basis functions through iterative approximation of NIRS features. Therefore, AFD can more effectively reconstruct the original NIRS data. In other words, AFD can more effectively obtain NIRS information. However, for the same NIRS data, the number of AFD-based features is much smaller than that of FFT-based and CWT-based features. Consequently, when evaluated as individual features, AFD-based features demonstrate weaker regression performance than either FFT-based or CWT-based features in modeling scenarios with a single feature.

## 4. Conclusions

This article uses a near-infrared spectrometer to acquire NIRS data from the powder samples of HSGT. The raw NIRS undergo preprocessing using five distinct methods, namely SG, MSC, SNV, MA and DT. Subsequently, FD and SD are applied to these preprocessed NIRS to generate the datasets with data processing. For four key substance indicators, such as theanine, tea polyphenols, soluble sugar, and water extract, the NIRS features are extracted by using three advanced algorithms, namely CARS, BC and AFD. The extracted features are subsequently employed to develop PLSR and RR models across single, dual, and triple features. For comprehensive comparison, FFT-based and CWT-based features combined with both BC and CARS are used to construct the estimation models. Key findings demonstrate that the CARS + BC + AFD based model yielded optimal predictive performance across all four substance indicators. To ensure statistical robustness, all reported results represent the average of 50 independent Monte Carlo simulation trials, effectively minimizing random sampling effects. Among them, for the theanine content, the RR estimation model is the best, with $R_{\mathrm{train}}^{2}=0.934$, $R_{\mathrm{validate}}^{2}=0.770$, NRMSE=0.139, RPD=2.175. For the tea polyphenol content, the RR estimation model is optimal, $R_{\mathrm{train}}^{2}=0.762$, $R_{\mathrm{validate}}^{2}=0.606$, NRMSE=0.180, RPD=1.671. For the soluble sugar content, the RR estimation model is also optimal, with $R_{\mathrm{train}}^{2}=0.980$, $R_{\mathrm{validate}}^{2}=0.805$, NRMSE=0.137, RPD=2.255. The PLSR estimation model for the water extract content is optimal, with $R_{\mathrm{train}}^{2}=0.674$, $R_{\mathrm{validate}}^{2}=0.561$, NRMSE=0.194, RPD=1.615. The experimental results not only demonstrate the effectiveness of using multiple feature variables to construct a model for estimating the substances’ content of tea, but also the performance-enhancing effect of AFD-based features. This study establishes a rapid and accurate method for detecting the substance content of HSGT, providing a scientific reference for its quality monitoring.

## Figures and Tables

**Table 1:** Spectral index.

Spectral Index	Theanine			Tea Polyphenols			Soluble Sugar			Water Extract		
	Preprocessing Methods	Calculation Formula	PCC	Preprocessing Methods	Calculation Formula	PCC	Preprocessing Methods	Calculation Formula	PCC	Preprocessing Methods	Calculation Formula	PCC
SR	DT-RAW	$R_{4501}/R_{9260}$	0.313	DT-RAW	$R_{4011}/R_{4810}$	0.332	MA-RAW	$R_{4370}/R_{10001}$	0.398	MSC-RAW	$R_{4189}/R_{7062}$	0.314
NSR	SNV-RAW	$\frac{R_{4073}/R_{6121}-1}{R_{4073}/R_{6121}+1}$	0.401	MA-RAW	$\frac{R_{4127}/R_{4686}-1}{R_{4127}/R_{4686}+1}$	0.455	MA-FD	$\frac{R_{4327}/R_{9827}-1}{R_{4327}/R_{9827}+1}$	0.664	SNV-RAW	$\frac{R_{4551}/R_{7020}-1}{R_{4551}/R_{7020}+1}$	0.384
DSI	DT-RAW	$R_{4000}-R_{8628}$	0.321	DT-SD	$R_{4000}-R_{6942}$	0.241						
NDSI	SNV-RAW	$\frac{R_{4073}-R_{6121}}{R_{4073}+R_{6121}}$	0.401	MA-RAW	$\frac{R_{4127}-R_{4686}}{R_{4127}+R_{4686}}$	0.455	MA-SD	$\frac{R_{4509}-R_{9824}}{R_{4509}+R_{9824}}$	0.664	SNV-RAW	$\frac{R_{4551}-R_{7020}}{R_{4551}+R_{7020}}$	0.384
GDSI	SNV-SD	$\frac{R_{4142}^{2}-R_{5022}}{R_{4142}^{2}+R_{5022}}$	0.434	MA-FD	$\frac{R_{4131}^{2}-R_{6912}}{R_{4131}^{2}+R_{6912}}$	0.449	MSC-RAW	$\frac{R_{4147}^{2}-R_{7467}}{R_{4147}^{2}+R_{7467}}$	0.421	SNV-RAW	$\frac{R_{4192}^{2}-R_{8960}}{R_{4192}^{2}+R_{8960}}$	0.386
TNDSI	SNV-SD	$\frac{\sqrt{R_{4293}-R_{9573}}}{R_{4273}+R_{9573}+0.5}$	0.500	MA-RAW	$\frac{\sqrt{R_{4532}-R_{4686}}}{R_{4532}+R_{4686}+0.5}$	0.453	MA-SD	$\frac{\sqrt{R_{4509}-R_{9824}}}{R_{4509}+R_{9824}+0.5}$	0.648	MSC-RAW	$\frac{\sqrt{R_{4034}-R_{7062}}}{R_{4034}+R_{7062}+0.5}$	0.384

**Table 2:** Optimal band ranges selected by different studies for the four indicators.

Indicator	Other Studies		This Study (${\mathrm{cm}}^{-1}$)
	Types of Tea	Band Ranges (${\mathrm{cm}}^{-1}$)	
Theanine	Green tea [47]	$\left[4246.7,\;5450.1\right]$, $\left[6800.1,\;7502\right]$	$\left[4007,\;5004\right]$, $\left[7065,\;9947\right]$
Tea polyphenols	Green tea [47]	$\left[5446.2,\;6101.9\right]$	$\left[4177,\;5827\right]$, [8800, 10,000]
Soluble sugar	Green tea [48]	$\left[7498.2,\;9997.7\right]$	$\left[4134,\;5480\right]$, $\left[6757,\;9993\right]$
Water extract	Oolong tea [49]	$\left[4246,\;4990\right]$	$\left[4320,\;5200\right]$

**Table 3:** Results of the optimal estimation model for four indicators of HSGT under difference features.

Indicator	Preprocessing Methods	Features	Model	$R_{\mathrm{train}}^{2}$	$R_{\mathrm{validate}}^{2}$	NRMSE	RPD
Theanine	MSC-SD	CARS	RR	0.914	0.735	0.140	2.157
	$R_{T}$	BC	PLSR	0.456	0.416	0.211	1.390
	SG-FD	AFD	RR	0.503	0.364	0.235	1.482
	MSC-SD	CARS + BC	RR	0.915	0.741	0.140	2.144
	MSC-SD	CARS + AFD	RR	0.926	0.780	0.137	2.204
	DT-RAW	BC + AFD	PLSR	0.624	0.544	0.197	1.511
	MSC-SD	CARS + BC + AFD	RR	0.934	0.770	0.139	2.175
Tea polyphenols	DT-FD	CARS	RR	0.702	0.541	0.186	1.563
	$R_{\mathrm{Tp}}$	BC	RR	0.426	0.351	0.213	1.330
	MA-FD	AFD	PLSR	0.626	0.513	0.202	1.567
	DT-FD	CARS + BC	RR	0.725	0.526	0.185	1.520
	MA-SD	CARS + AFD	RR	0.722	0.582	0.171	1.673
	MA-SD	BC + AFD	RR	0.712	0.476	0.206	1.599
	DT-FD	CARS + BC + AFD	RR	0.762	0.606	0.180	1.671
Soluble sugar	RAW-SD	CARS	RR	0.940	0.789	0.127	2.293
	$R_{\mathrm{Ss}}$	BC	RR	0.544	0.494	0.197	1.559
	MA-FD	AFD	PLSR	0.607	0.520	0.191	1.547
	RAW-SD	CARS + BC	RR	0.937	0.792	0.124	2.334
	RAW-SD	CARS + AFD	RR	0.957	0.802	0.129	2.384
	MA-FD	BC + AFD	RR	0.700	0.577	0.179	1.630
	RAW-SD	CARS + BC + AFD	RR	0.980	0.805	0.137	2.255
Water extract	MA-FD	CARS	PLSR	0.571	0.544	0.199	1.579
	$R_{\mathrm{We}}$	BC	PLSR	0.200	0.140	0.255	1.166
	MA-SD	AFD	PLSR	0.563	0.507	0.185	1.578
	DT-SD	CARS + BC	PLSR	0.580	0.493	0.193	1.617
	MA-FD	CARS + AFD	RR	0.708	0.488	0.202	1.545
	MA-FD	BC + AFD	RR	0.671	0.444	0.217	1.585
	MA-FD	CARS + BC + AFD	PLSR	0.674	0.561	0.194	1.615

**Table 4:** Optimal estimation model results for four indicators of HSGT under the comparative features.

Indicator	Preprocessing Methods	Features	Model	$R_{\mathrm{train}}^{2}$	$R_{\mathrm{validate}}^{2}$	NRMSE	RPD
Theanine	SNV-RAW	FFT	PLSR	0.547	0.399	0.218	1.367
	SNV-FD	Bump	RR	0.574	0.392	0.215	1.386
	MSC-SD	CARS + FFT	RR	0.915	0.744	0.140	2.092
	MSC-SD	CARS + Amor	RR	0.941	0.734	0.140	2.156
	SNV-RAW	BC + FFT	RR	0.662	0.356	0.223	1.322
	SG-FD	BC + Bump	RR	0.635	0.465	0.202	1.471
	MSC-SD	CARS + BC + FFT	RR	0.917	0.746	0.142	2.154
	MSC-SD	CARS + BC + Amor	RR	0.938	0.759	0.131	2.201
Tea polyphenols	MA-SD	FFT	RR	0.682	0.457	0.199	1.456
	MA-FD	Bump	RR	0.593	0.496	0.191	1.540
	DT-FD	CARS + FFT	RR	0.770	0.425	0.202	1.497
	MA-SD	CARS + Amor	RR	0.727	0.448	0.194	1.457
	SNV-RAW	BC + FFT	RR	0.675	0.446	0.204	1.454
	MA-FD	BC + Morse	RR	0.646	0.484	0.189	1.563
	DT-FD	CARS + BC + FFT	RR	0.794	0.544	0.176	1.596
	DT-FD	CARS + BC + Bump	RR	0.788	0.520	0.183	1.544
Soluble sugar	MA-FD	FFT	RR	0.764	0.561	0.195	1.597
	MD-FD	Bump	RR	0.641	0.517	0.192	1.507
	RAW-SD	CARS + FFT	RR	0.956	0.809	0.128	2.431
	RAW-SD	CARS + Morse	RR	0.953	0.765	0.133	2.201
	MA-RAW	BC + FFT	RR	0.747	0.487	0.203	1.525
	DT-FD	BC + Morse	RR	0.702	0.539	0.184	1.537
	RAW-SD	CARS + BC + FFT	RR	0.951	0.797	0.124	2.355
	RAW-SD	CARS + BC + Amor	RR	0.943	0.772	0.130	2.243
Water extract	MA-FD	FFT	RR	0.765	0.530	0.185	1.696
	MA-RAW	Bump	RR	0.640	0.529	0.190	1.540
	MA-FD	CARS + FFT	RR	0.750	0.537	0.196	1.559
	MA-SD	CARS + Bump	PLSR	0.604	0.521	0.191	1.596
	MA-FD	BC + FFT	RR	0.776	0.541	0.202	1.588
	MA-SD	BC + Amor	RR	0.628	0.447	0.201	1.500
	MA-FD	CARS + BC + FFT	RR	0.774	0.557	0.183	1.610
	MA-FD	CARS + BC + Morse	RR	0.604	0.535	0.195	1.559

## Data Availability

The data presented in this study are available on request from the corresponding author due to restriction.

## Supplementary Materials

The following supporting information can be downloaded at: https://www.mdpi.com/article/10.3390/foods15030531/s1, Table S1: Results of the optimal PLSR model for four indicators of HSGT. Table S2: Results of the optimal RR model for four indicators of HSGT. Table S3: Optimal PLSR model results for four indicators under the comparative features. Table S4: Optimal RR model results for four indicators under the comparative features.

## Abbreviations

The following abbreviations are used in this manuscript:

AFD	Adaptive Fourier decomposition
BC	Band combination
CARS	Competitive adaptive re-weighted sampling
CWT	Continuous wavelet transform
DT	Detrended terms
DSI	Difference spectral index
FD	First-order derivative
FFT	Fast Fourier transform
GDSI	Generalized difference spectral index
HSGT	Hakka stir-fried green tea
MA	Moving average
MSC	Multivariate scattering correction
NDSI	Normalized difference spectral index
NIRS	Near-infrared spectroscopy
NRMSE	Normalized root-mean-square error
NSR	Normalized sample ratio
PCC	Pearson correlation coefficient
PLSR	Partial least squares regression
REE	Relative energy error
RPD	Ratio of performance to deviation
RR	Ridge regression
SD	Second-order derivative
SG	Savitzky-Golay smoothing
SR	Sample ratio
SNV	Standard normal variate
TNDSI	Transformed normalized difference spectral index
