# Supplementary material for: Prediction of Quality Substance Content of Hakka Stir-Fried Green Tea Based on Multiple Features of Near-Infrared Spectroscopy
Source: Foods. 2026 Feb 3;15(3):531. doi: 10.3390/foods15030531 (PMC12896664; doi:10.3390/foods15030531)
Supplement: Supplementary file 1 [file foods-15-00531-s001.zip › foods-4088852-supplementary.pdf]

Table S1. Results of the optimal PLSR model for four indicators of HSGT.

Indicator	Preprocessing methods	Features	R^2_{train}	$R^2_{\text{validation}}$	*NRMSE*	*RPD*
Theanine	MSC-SD	CARS	0.87562	0.73146	0.13873	2.1412
	R_T	BC	0.29841	0.18622	0.2509	1.2303
	SG-FD	AFD	0.44589	0.35767	0.21673	1.3254
	MSC-SD	CARS-BC	0.87658	0.72802	0.14495	2.0968
	MSC-SD	CARS-AFD	0.87622	0.71649	0.14798	2.044
	DT-RAW	BC-AFD	0.62077	0.54396	0.19732	1.5108
	MSC-SD	BC-CARS-AFD	0.88244	0.71965	0.14593	2.0923
Tea polyphenols	MA-SD	CARS	0.62073	0.57766	0.16123	1.7465
	RAW-RAW	BC	0.40986	0.3442	0.21724	1.3321
	MA-SD	AFD	0.47181	0.36329	0.21109	1.3403
	DT-FD	CARS-BC	0.66076	0.54398	0.17739	1.6413
	MA-SD	CARS-AFD	0.65632	0.58853	0.16777	1.8066
	SNV-RAW	BC-AFD	0.57915	0.43693	0.20016	1.444
	MA-SD	BC-CARS-AFD	0.68741	0.58138	0.16685	1.7468
Soluble sugar	RAW-SD	CARS	0.90505	0.78268	0.13162	2.4043
	RAW-RAW	BC	0.52996	0.52111	0.18718	1.5035
	MA-FD	AFD	0.60712	0.51781	0.19071	1.5471
	RAW-SD	CARS-BC	0.8981	0.77125	0.13621	2.3046
	RAW-SD	CARS-AFD	0.91107	0.75239	0.13647	2.1979
	DT-SD	BC-AFD	0.59483	0.52436	0.19318	1.618
	RAW-SD	BC-CARS-AFD	0.91308	0.76473	0.13251	2.256
Water extract	MA-FD	CARS	0.57069	0.54426	0.19871	1.5794
	RAW-RAW	BC	0.20014	0.14027	0.25468	1.1655
	MA-FD	AFD	0.56324	0.50731	0.18463	1.5776
	MA-SD	CARS-BC	0.57972	0.49323	0.19259	1.6168
	MA-SD	CARS-AFD	0.58023	0.45463	0.20577	1.4515
	MA-FD	BC-AFD	0.55086	0.45877	0.2138	1.4556
	MA-SD	BC-CARS-AFD	0.67361	0.56103	0.1941	1.6147

Table S2. Results of the optimal RR model for four indicators of HSGT

Indicator	Preprocessing methods	Features	R^2_{train}	$R^2_{\text{validation}}$	*NRMSE*	*RPD*
Theanine	MSC-SD	CARS	0.91417	0.73482	0.14018	2.1568
	RAW-RAW	BC	0.29991	0.17259	0.25308	1.2131
	SG-FD	AFD	0.50291	0.36353	0.23488	1.4817
	MSC-SD	CARS-BC	0.91553	0.74058	0.1397	2.1443
	MSC-SD	CARS-AFD	0.92348	0.74685	0.14763	2.2001

	SG-FD	BC-AFD	0.56894	0.40801	0.21096	1.3706
	MSC-SD	CARS-BC-AFD	0.93035	0.77041	0.13191	2.1506
Tea polyphenols	DT-FD	CARS	0.70194	0.5406	0.18642	1.5626
	RAW-RAW	BC	0.42598	0.35123	0.2131	1.3303
	MA-SD	AFD	0.52961	0.36275	0.21666	1.3642
	DT-FD	CARS-BC	0.72474	0.52562	0.18492	1.5200
	SG-SD	CARS-AFD	0.72646	0.58169	0.17117	1.6728
	MA-SD	BC-AFD	0.71188	0.47577	0.20598	1.5987
	DT-FD	BC-CARS-AFD	0.76162	0.60596	0.18031	1.6707
Soluble sugar	RAW-SD	CARS	0.93981	0.78876	0.12687	2.2926
	RAW-RAW	BC	0.54395	0.49391	0.19743	1.5589
	MA-FD	AFD	0.5214	0.39526	0.22975	1.3818
	RAW-SD	CARS-BC	0.93706	0.7916	0.12427	2.3336
	RAW-SD	CARS-AFD	0.95733	0.80165	0.12903	2.3838
	DT-SD	BC-AFD	0.69971	0.57723	0.17886	1.6302
	RAW-SD	BC-CARS-AFD	0.97978	0.80477	0.13659	2.2546
Water extract	DT-SD	CARS	0.6383	0.12508	0.26309	1.1825
	RAW-RAW	BC	0.22417	0.06210	0.28591	1.0894
	MA-FD	AFD	0.52189	0.33074	0.23998	1.3153
	DT-SD	CARS-BC	0.67286	0.29216	0.24549	1.2703
	RAW-SD	CARS-AFD	0.70823	0.48832	0.20186	1.54497
	MA-FD	BC-AFD	0.67121	0.4443	0.21688	1.5848
	DT-SD	BC-CARS-AFD	0.74302	0.45363	0.2533	1.4362

Table S3. Optimal PLSR model results for four indicators under the comparative features.

Indicator	Preprocessing methods	Features	R^2_{train}	$R^2_{validation}$	*NRMSE*	*RPD*
Theanine	SNV-RAW	FFT	0.52856	0.40242	0.21728	1.3686
	MSC-SD	CARS-FFT	0.87228	0.72197	0.14773	2.0265
	SNV-RAW	BC-FFT	0.51674	0.33871	0.2268	1.3212
	MSC-SD	CARS-BC-FFT	0.87152	0.71055	0.15072	1.9907
	DT-RAW	Morse	0.44529	0.38213	0.21907	1.343
	MSC-SD	CARS-Morse	0.87701	0.69837	0.14809	1.9874
	RAW-FD	BC-Morse	0.49945	0.32265	0.22667	1.3011
	MSC-SD	CARS-BC-Morse	0.87449	0.7106	0.1475	2.0158
	SNV-FD	Amor	0.43997	0.35428	0.22817	1.3225
	MSC-SD	CARS-Amor	0.88136	0.72399	0.14436	2.0599
	RAW-FD	BC-Amor	0.50344	0.35599	0.22645	1.3078
	MSC-SD	CARS-BC-Amor	0.87918	0.71915	0.14572	2.0342
	MSC-FD	Bump	0.50449	0.35779	0.22485	1.3497
	MSC-SD	CARS-Bump	0.86912	0.71584	0.14583	2.0505

	RAW-FD	BC-Bump	0.55797	0.40173	0.21184	1.3509
	MSC-SD	CARS-BC-Bump	0.86827	0.73488	0.13735	2.1196
Tea polyphenols	SNV-RAW	FFT	0.57756	0.49732	0.18662	1.5718
	MSC-SD	CARS-FFT	0.66222	0.42355	0.20507	1.4366
	SNV-RAW	BC-FFT	0.6097	0.49929	0.18902	1.526
	MSC-SD	CARS-BC-FFT	0.71115	0.537	0.17181	1.6116
	MA-FD	Morse	0.4986	0.43827	0.20297	1.4336
	DT-FD	CARS-Morse	0.61446	0.3964	0.20772	1.3712
	MSC-SD	BC-Morse	0.57466	0.42139	0.21281	1.4224
	DT-FD	CARS-BC-Morse	0.68994	0.46945	0.18661	1.4752
	MA-FD	Amor	0.47061	0.40459	0.20759	1.4424
	DT-FD	CARS-Amor	0.61138	0.40627	0.20817	1.4274
	SNV-SD	BC-Amor	0.54823	0.49767	0.19027	1.4895
	DT-FD	CARS-BC-Amor	0.68059	0.50011	0.18138	1.5055
	MA-FD	Bump	0.53193	0.49144	0.18949	1.5464
	DT-FD	CARS-Bump	0.62571	0.43955	0.20013	1.4221
	MA-FD	BC-Bump	0.57131	0.48843	0.18813	1.5431
	DT-FD	CARS-BC-Bump	0.7102	0.48622	0.19228	1.4696
Soluble sugar	RAW-SD	CARS	0.90505	0.78268	0.13162	2.4043
	RAW-RAW	BC	0.52996	0.52111	0.18718	1.5035
	MA-FD	AFD	0.60712	0.51781	0.19071	1.5471
	RAW-SD	CARS-BC	0.8981	0.77125	0.13621	2.3046
	RAW-SD	CARS-AFD	0.91107	0.75239	0.13647	2.1979
	DT-SD	BC-AFD	0.59483	0.52436	0.19318	1.618
	RAW-SD	BC-CARS-AFD	0.91308	0.76473	0.13251	2.256
	MA-FD	FFT	0.59793	0.47568	0.20341	1.5004
	RAW-SD	CARS-FFT	0.92261	0.73928	0.13851	2.1625
	SNV-RAW	BC-FFT	0.61471	0.52437	0.2007	1.574
	RAW-SD	CARS-BC-FFT	0.91601	0.78804	0.12412	2.2821
	MA-FD	Morse	0.56562	0.5001	0.19874	1.5611
	RAW-SD	CARS-Morse	0.91194	0.73712	0.14946	2.165
	SNV-SD	BC-Morse	0.61376	0.51565	0.19104	1.5223
	RAW-SD	CARS-BC-Morse	0.90315	0.74605	0.13719	2.2611
	MA-FD	Amor	0.52967	0.48142	0.20147	1.559
Water extract	MA-FD	FFT	0.60624	0.51412	0.19917	1.5616
	MA-SD	CARS-FFT	0.6161	0.49934	0.19599	1.5087
	MA-FD	BC-FFT	0.60057	0.49807	0.21001	1.5261
	MA-SD	CARS-BC-FFT	0.60536	0.48374	0.19773	1.5305
	MA-FD	Morse	0.57598	0.49133	0.20778	1.5221
	MA-FD	CARS-Morse	0.60304	0.51815	0.19573	1.529
	MA-FD	BC-Morse	0.58678	0.50699	0.19477	1.6052
	MA-FD	CARS-BC-Morse	0.60438	0.53505	0.19465	1.5586
	MA-FD	Amor	0.54272	0.43746	0.21456	1.4951

	MA-SD	CARS-Amor	0.58232	0.43355	0.22192	1.4251
	MA-FD	BC-Amor	0.54994	0.48562	0.19768	1.5167
	MA-FD	CARS-BC-Amor	0.5794	0.47334	0.20837	1.4795
	MA-RAW	Bump	0.58559	0.51444	0.19328	1.5645
	MA-SD	CARS-Bump	0.60447	0.52059	0.19092	1.5964
	MA-RAW	BC-Bump	0.5799	0.43812	0.20536	1.4632
	MA-FD	CARS-BC-Bump	0.61633	0.48863	0.19903	1.558

Table S4. Optimal RR model results for four indicators under the comparative features.

Indicator	Preprocessing methods	Features	R^2_{train}	$R^2_{validation}$	*NRMSE*	*RPD*
Theanine	SNV-RAW	FFT	0.5614	0.35626	0.22309	1.3204
	MSC-SD	CARS-FFT	0.91559	0.74383	0.13951	2.0919
	SNV-RAW	BC-FFT	0.66187	0.35552	0.22277	1.3216
	MSC-SD	CARS-BC-FFT	0.91686	0.74618	0.14196	2.1538
	DT-FD	Morse	0.54521	0.23111	0.25336	1.2104
	MSC-SD	CARS-Morse	0.93373	0.73803	0.14926	2.091
	DT-FD	BC-Morse	0.5999	0.36906	0.21874	1.3687
	MSC-SD	CARS-BC-Morse	0.93056	0.72591	0.14597	2.0823
	SNV-SD	Amor	0.52198	0.36443	0.22498	1.3482
	MSC-SD	CARS-Amor	0.94074	0.73394	0.14018	2.1558
	DT-FD	BC-Amor	0.56985	0.29695	0.23998	1.2953
	MSC-SD	CARS-BC-Amor	0.93798	0.75886	0.13127	2.2009
	SNV-FD	Bump	0.57421	0.39165	0.21461	1.3864
	MSC-SD	CARS-Bump	0.92116	0.72318	0.14826	2.0396
	SG-FD	BC-Bump	0.63511	0.46474	0.20198	1.4709
	MSC-SD	CARS-BC-Bump	0.91964	0.7209	0.14925	2.0239
Tea polyphenols	MA-SD	FFT	0.64082	0.38513	0.20831	1.3723
	DT-FD	CARS-FFT	0.73955	0.43986	0.19961	1.4208
	SNV-RAW	BC-FFT	0.67467	0.44578	0.20415	1.4536
	DT-FD	CARS-BC-FFT	0.77218	0.51997	0.1885	1.524
	MA-FD	Morse	0.58099	0.42907	0.20388	1.4323
	SG-SD	CARS-Morse	0.72887	0.42684	0.20328	1.4283
	MA-FD	BC-Morse	0.64624	0.48428	0.18872	1.5627
	DT-FD	CARS-BC-Morse	0.77211	0.49642	0.19027	1.4963
	MA-SD	Amor	0.54191	0.30667	0.21762	1.3656
	MA-SD	CARS-Amor	0.72666	0.44816	0.1941	1.4573
	MA-SD	BC-Amor	0.61716	0.39488	0.20668	1.4404
	DT-FD	CARS-BC-Amor	0.75245	0.45255	0.20461	1.489
	MA-FD	Bump	0.59283	0.4955	0.19114	1.5394
	DT-FD	CARS-Bump	0.72521	0.4236	0.20063	1.4032

	MA-FD	BC-Bump	0.64811	0.37876	0.2106	1.4013
	DT-FD	CARS-BC-Bump	0.78762	0.51988	0.18289	1.5435
Soluble sugar	MA-FD	FFT	0.76378	0.56123	0.19464	1.5969
	RAW-SD	CARS-FFT	0.9563	0.80851	0.12786	2.4311
	MA-FD	BC-FFT	0.74729	0.48672	0.20309	1.5254
	RAW-SD	CARS-BC-FFT	0.95089	0.79674	0.12375	2.3554
	MA-FD	Morse	0.63401	0.52304	0.1962	1.6079
	RAW-SD	CARS-Morse	0.95303	0.76453	0.13329	2.2013
	MSC-SD	BC-Morse	0.68451	0.50276	0.19401	1.4919
	RAW-SD	CARS-BC-Morse	0.94872	0.77649	0.12841	2.272
	MA-FD	Amor	0.60875	0.4558	0.21402	1.4504
	RAW-SD	CARS-Amor	0.94962	0.76435	0.13336	2.1955
	SNV-FD	BC-Amor	0.6765	0.46883	0.19963	1.4866
	RAW-SD	CARS-BC-Amor	0.94307	0.77225	0.12969	2.2429
	MA-FD	Bump	0.64059	0.51739	0.192	1.5071
	RAW-SD	CARS-Bump	0.9518	0.72068	0.15253	2.1803
	DT-RAW	BC-Bump	0.70244	0.53867	0.18364	1.5373
	RAW-SD	CARS-BC-Bump	0.94614	0.76134	0.13828	2.1671
Water extract	MA-FD	FFT	0.76512	0.52987	0.18544	1.6963
	MA-FD	CARS-FFT	0.75051	0.53673	0.19595	1.5588
	MA-FD	BC-FFT	0.77632	0.54089	0.2017	1.5877
	MA-FD	CARS-BC-FFT	0.77404	0.55733	0.18283	1.6099
	MA-FD	Morse	0.64087	0.49729	0.20416	1.5134
	DT-SD	CARS-Morse	0.73797	0.17179	0.26757	1.2371
	MA-FD	BC-Morse	0.65183	0.4775	0.21205	1.526
	DT-SD	CARS-BC-Morse	0.76511	0.24336	0.25587	1.2893
	MA-FD	Amor	0.62109	0.50387	0.20304	1.5153
	DT-SD	CARS-Amor	0.71289	0.17866	0.24288	1.2774
	MA-FD	BC-Amor	0.62782	0.44705	0.2006	1.5000
	DT-SD	CARS-BC-Amor	0.74154	0.24717	0.23352	1.3073
	MA-RAW	Bump	0.63975	0.52922	0.19012	1.5402
	DT-SD	CARS-Bump	0.74388	0.19741	0.2645	1.1896
	MA-FD	BC-Bump	0.65382	0.50675	0.20087	1.5153
	RAW-SD	CARS-BC-Bump	0.7626	0.32009	0.23054	1.3006
